# Rituximab-containing therapy for cold agglutinin disease: a retrospective study of 16 patients

**DOI:** 10.1038/s41598-020-69465-2

**Published:** 2020-07-29

**Authors:** Ming-nan Jia, Yu Qiu, Yan-yan Wu, Hao Cai, Dao-bin Zhou, Xin-xin Cao, Jian Li

**Affiliations:** Department of Haematology, Peking Union Medical College Hospital, Chinese Academy of Medical Sciences & Peking Union Medical College, Beijing, 100730 China

**Keywords:** Anaemia, Chemotherapy

## Abstract

Cold agglutinin disease (CAD) is a rare form of autoimmune haemolytic anaemia, and because of its rareness, there is no standard treatment for CAD patients. We retrospectively analysed the response to rituximab-containing therapy in CAD patients at our hospital. All patients received rituximab-containing therapy for at least 1 month. A total of 16 patients (11 males and 5 females) were included. The median age at the onset of the disease was 63.5 years (range 41–79). Most patients had manifestations including anaemia (81.3%) or cold-induced circulatory symptoms (75.0%). The median haemoglobin level was 72 g/L (range 29–101), and the median cold agglutinin titre was 1,024 (range 64–2,048). Thirteen of 16 patients (81%) responded to the therapy. Responders achieved a median increase in haemoglobin levels of 45 g/L. Grade 3–4 neutropenia occurred in 3 patients (19%), but only 1 (6%) of them experienced infection. Anaphylaxis related to rituximab occurred in 1 patient. During follow-up, five patients experienced relapse, and two patients died. The estimated median progression-free survival was 36 months, and median overall survival was not yet reached. In conclusion, A rituximab-based therapy in accordance with individual patient characteristics may be a reasonable choice for CAD patients.

## Introduction

Cold agglutinin disease (CAD) is an autoimmune haemolytic anaemia without other underlying diseases such as aggressive lymphoma, overt malignancies, or specific infections^1,2^. CAD is associated with a clonal lymphoproliferative disorder (LPD), producing monoclonal cold agglutinins (CA), usually immunoglobin (Ig) M autoantibodies^2,3^. CAs bind to type I carbohydrate antigen on the erythrocyte surface below normal central body temperatures, resulting in complement-mediated, classical pathway-dependent autoimmune haemolysis^1,4,5^. Extravascular destruction of C3b-coated erythrocytes is considered to be the predominant mechanism of erythrocyte breakdown in CAD patients^6^. As a rare disease, CAD accounts for approximately 15% of autoimmune haemolytic anaemias^2^. The most common clinical manifestations include anaemia, cold-induced acrocyanosis and Raynaud phenomena^1^.

At present, there is no approved treatment for CAD, and conducting randomized studies with sufficient statistical power will be difficult given the rareness of CAD. Non-pharmacological management, such as keeping warm, warm liquid infusions, and erythrocyte transfusions, is still a choice^7–9^. Reasonable criteria for beginning drug therapy should include symptomatic anaemia, transfusion dependence, and disabling circulatory symptoms^1^. Corticosteroids and other unspecific immune suppression have failed to demonstrate an acceptable effect^3,8,10–12^. The first breakthrough, rituximab monotherapy, 375 mg/m^2^ once a week for four weeks, resulted in a response rate of 50–60%, but with a response duration of only 11 months^9,13–15^. Subsequently, a combination of rituximab with fludarabine had a satisfactory response rate of 76%, with a complete remission rate of 21% and a long response duration, but toxicity is a concern^16^. In 2017, Berentsen et al. published their results in the use of bendamustine-rituximab therapy, which resulted in a similar response rate of 71%, with an even higher complete remission rate of 40% and sustained remissions, and the safety profile appeared more amenable than the fludarabine-rituximab combination^17^. Until recently, most effective therapies have been directed at pathogenic B-cells, and the response rate and response duration need further improvement; bendamustine-rituximab therapy or rituximab monotherapy should be considered first-line treatments^15,18^. However, none of these studies have been confirmed sufficiently.

Given the limitations in therapy choices for CAD, we have retrospectively analysed the efficacy of rituximab-containing therapies in the treatment of CAD patients at our hospital.

## Methods

### Patients

Patients diagnosed with CAD and who received any rituximab-containing therapy for at least 1 month between January 2012 and September 2019 at the Peking Union Medical College Hospital, were included in this retrospective study. The diagnosis of CAD was as previously described^1^: (i) Chronic haemolysis; (ii) Coombs testing strongly positive, especially for C3d; (iii) CA titre ≥ 64 at 4 °C. Patients associated with an aggressive lymphoma or infection were ineligible for the study. Clinical data, including age, sex, disease duration, clinical manifestations, physical examination results, laboratory test results, treatment and survival, were collected for all patients.

We detected *MYD88*^L265P^ and *CXCR4*^S338X^ mutations by real-time allele-specific oligonucleotide polymerase chain reaction using unsorted bone marrow samples before January 2018, as previously described^19^. Since January 2018, a next‐generation sequencing assay covering 69 genes was used to investigate recurrently mutated genes. Read pairs were aligned to Refseq hg19 (downloaded from UCSC Genome Browser, URLs) by Burrows–Wheeler Aligner (BWA) version 0.7.13-r1126. Samtools version 1.3 was used to generate chromosomal coordinate-sorted bam files. The mean depth of each sample was 1,000 ×, with an average of 5% of the target sequence being covered sufficiently deeply for variant calling. Samtools mpileup was applied for SNV/indel calling and filter workflow.

Waldenström macroglobulinemia (WM), splenic marginal zone lymphoma, and chronic lymphocytic lymphoma were diagnosed according to the World Health Organization classification^20^.

### Treatment

All patients received one of the following rituximab-containing regimens: rituximab monotherapy (with or without prednisone, 375 mg/m^2^ once a week for four weeks), R-CHOP (rituximab, cyclophosphamide, doxorubicin, vincristine, and prednisone), R-CVP (rituximab, cyclophosphamide, vincristine, and prednisone), DRC (dexamethasone, rituximab and cyclophosphamide), RFC (rituximab, fludarabine and cyclophosphamide).

### Response criteria

The response criteria were referenced from previously published papers^1^. The criteria for complete response (CR) were a disappearance of clinical symptoms of CAD, an absence of anaemia, no signs of haemolysis, and no monoclonal serum protein and no signs of clonal lymphoproliferation as assessed by bone marrow histology, immunohistochemistry, and flow cytometry. Partial response (PR) was defined as a stable increase in haemoglobin (Hgb) levels by at least 20 g/L or to the normal range, a reduction of serum IgM levels by at least 50% of the initial level or to the normal range, improvement of clinical symptoms and transfusion independence. Any patient not meeting the criteria for CR or PR was labelled no response (NR).

Overall survival (OS) was calculated from the date of initiation of treatment to the date of death or last follow-up. Progression-free survival (PFS) was defined as initiation of treatment until relapse, death or last follow-up. Relapse was defined as the need for re-treatment, as mentioned above. The final follow-up date was Sep 30, 2019.

### Statistics

Median values were calculated for variables with non-normal distributions. The statistical significance of differences between paired continuous data was calculated with the Wilcoxon signed-rank test. OS and PFS were estimated according to Kaplan–Meier survival analysis. Statistical calculations were performed on a personal computer using SPSS version 24.0.0.0 software (SPSS Inc., Chicago, IL, USA). All figures were performed on a personal computer using GraphPad Prism version 8.0.0 for Windows, GraphPad Software, San Diego, California USA, www.graphpad.com.

### Ethical approval and informed consent

Written informed consent was obtained from all individual participants included in the study. The protocol was approved by the Peking Union Medical College Hospital Ethics Committee. This study was performed in accordance with the 1964 Declaration of Helsinki and its later amendments or comparable ethical standards.

## Results

### Baseline characteristics

Sixteen patients were included, 11 men and 5 women. The median age at the onset of clinical symptoms or anaemia was 63.5 years (range 41–79), and the median duration from the onset of clinical symptoms or anaemia until the time of diagnosis was 7.5 months (range 1–129). Detailed information for the patients included in this study is shown in Table 1. Cold-induced circulatory symptoms were found in 12 patients (75.0%), 13 patients (81.3%) had symptoms related to anaemia such as fatigue and weakness, and 5 patients (31.3%) had symptoms pertinent to haemolysis such as jaundice and haemoglobinuria. Five patients (31.3%) received erythrocyte transfusions before therapy. Twelve patients were diagnosed with indolent lymphoma, including 7 WM, 2 splenic marginal zone lymphoma, 1 chronic lymphocytic lymphoma and 2 indolent B-cell lymphomas that were unclassifiable according to the World Health Organization classification. At baseline, the median Hgb level was 72 g/L (range 29–101), the median reticulocyte percentage was 5.29% (range 1.02–11.38), the median total bilirubin was 30.4 μmol/L (range 6.2–154.2), the median indirect bilirubin was 21.0 μmol/L (range 2.2–85.0), and the median lactate dehydrogenase (LDH) was 280 U/L (range 124–517, normal range 0–250). The serum monoclonal Ig class was IgMκ in 15 patients (93.8%) and IgMλ in 1 patient. The median monoclonal immunoglobulin of 14 patients was 2.7 g/L (range 0.1–38.0), and the remaining 2 patients did not have monoclonal immunoglobulin tested at baseline. The median IgM level was 7.11 g/L (range 1.58–55.8). The results of Coombs testing were all positive; 13 patients had strong positive anti-C3 and anti-C3d antibodies, 1 was positive for anti-IgM antibodies, and the remaining 2 patients had unspecific results. All CA titres were ≥ 64 by definition, and the median CA titre was 1,024 (range 64–2,048). The *MYD88*^L265P^ and *CXCR4*^S338X^ mutations were tested for 4 patients by real-time allele-specific oligonucleotide polymerase chain reaction, and a next-generation sequencing assay was performed for the other 4 patients using their bone marrow. The *MYD88*^L265P^ mutation was shown in 2 patients (25%), and both were diagnosed with WM simultaneously. None of the patients showed the *CXCR4*^S338X^ mutation. Patient 5 and patient 16 showed mutations in *TP53* and *TET2,* respectively, by the next‐generation sequencing assay, and patient 5 was diagnosed with unclassifiable indolent B-cell lymphoma simultaneously.

**Table 1:** The baseline characteristics and treatment responses of patients.

No.	Sex/age, years	Disease duration, months	Underlying disease	Previous treatment	Hgb, g/L	Ret, %	Ibil, μmol/L	LDH, U/L	IFE	IgM, g/L	CA titre	MYD88 gene	Treatment	Courses including R	Response	PFS, m	OS, m
1	M/79	85	WM	Chlorambucil + P	63	11.83	17.2	396	IgMκ	8.80	1,024	NA	R + P	4	PR	3	5
2	F/71	4	WM	Newly diagnosed	39	9.06	41.5	358	IgMκ	5.30	2,048	NA	R	4	PR	62	79+
3	M/55	5	None	Newly diagnosed	82	8.40	15.7	232	IgMκ	4.87	64	NA	R	4	PR	12+	12+
4	M/64	4	None	P	74	3.62	21.5	275	IgMκ	5.41	1,024	wild type	R	4	CR	16+	16+
5	M/73	2	Unclassifiable indolent B-cell lymphoma	Newly diagnosed	72	6.95	26.2	311	IgMκ	4.39	256	wild type	R	4	PR	16+	16+
6	F/60	129	None	Chlorambucil + P	29	8.86	20.4	285	IgMκ	5.10	1,024	wild type	R	4	NR	1+	1+
7	M/41	3	SMZL	Newly diagnosed	101	3.46	24.8	517	IgMκ	13.00	2,048	NA	R-CHOP	8	PR	36	82+
8	M/63	105	CLL	Chlorambucil	78	5.29	7.4	370	IgMλ	9.57	1,024	NA	R-CVP	5	PR	12+	12+
9	M/69	9	Unclassifiable indolent B-cell lymphoma	P	41	1.02	85.0	174	IgMκ	1.58	128	NA	R-CVP	6	CR	33+	33+
10	F/61	63	WM	F + chlorambucil	78	1.89	6.6	124	IgMκ	33.87	1,024	MYD88^L265P^	DRC/RFC	2	PR	24+	24+
11	M/63	2	WM	Newly diagnosed	88	2.13	12.1	363	IgMκ	44.70	1,024	wild type	DRC/RFC	2	PR	3+	3+
12	F/49	106	WM	Newly diagnosed	76	3.97	2.2	218	IgMκ	55.80	2,048	wild type	DRC/RFC	7	PR	13	13
13	M/69	52	WM	Chlorambucil + P	72	2.63	23.4	244	IgMκ	2.37	512	NA	DRC	3	NR	40+	40+
14	M/76	1	SMZL	Newly diagnosed	52	7.31	36.4	426	IgMκ	9.95	2,048	NA	DRC	4	CR	15	36+
15	F/65	6	WM	Newly diagnosed	58	NA	6.5	211	IgMκ	47.48	64	MYD88^L265P^	DRC	5	PR	9	30+
16	M/52	62	None	Newly diagnosed	72	11.38	22.3	239	IgMκ	3.64	1,024	wild type	DRC	2	NR	2+	2+

*WM* Waldenström macroglobulinemia, *SMZL* splenic marginal zone lymphoma, *CLL* chronic lymphocytic lymphoma, *P* prednisone, *F* fludarabine, *Hgb* haemoglobin, *Ret* reticulocyte, *NA* not available, *Ibil* indirect bilirubin, *LDH* lactate dehydrogenase, *IFE* immunofixation electrophoresis, *Ig* immunoglobulin, *CA* cold agglutinin, *R* rituximab, *R-CHOP* rituximab, cyclophosphamide, doxorubicin, vincristine and prednisone, *R-CVP* rituximab, cyclophosphamide, vincristine, and prednisone, *DRC* dexamethasone, rituximab and cyclophosphamide, *RFC* rituximab, fludarabine, cyclophosphamide, + , the patient has not yet experienced relapse and (or) death.

### Treatment and response

Six patients received single-agent rituximab with or without prednisone, while the other 10 patients were treated with one of the rituximab-containing therapies: 1 R-CHOP, 2 R-CVP, 3 DRC/RFC combination and 4 DRC. The treatments resulted in 13 PRs (81%), of which 3 (19%) fulfilled all CR criteria except for the bone marrow examination (patients 4, 9, 14), and 3 NR (19%). The difference in the response rate between single-agent rituximab and the rituximab-containing therapies was not significant (P > 0.05).

**Figure 1:**
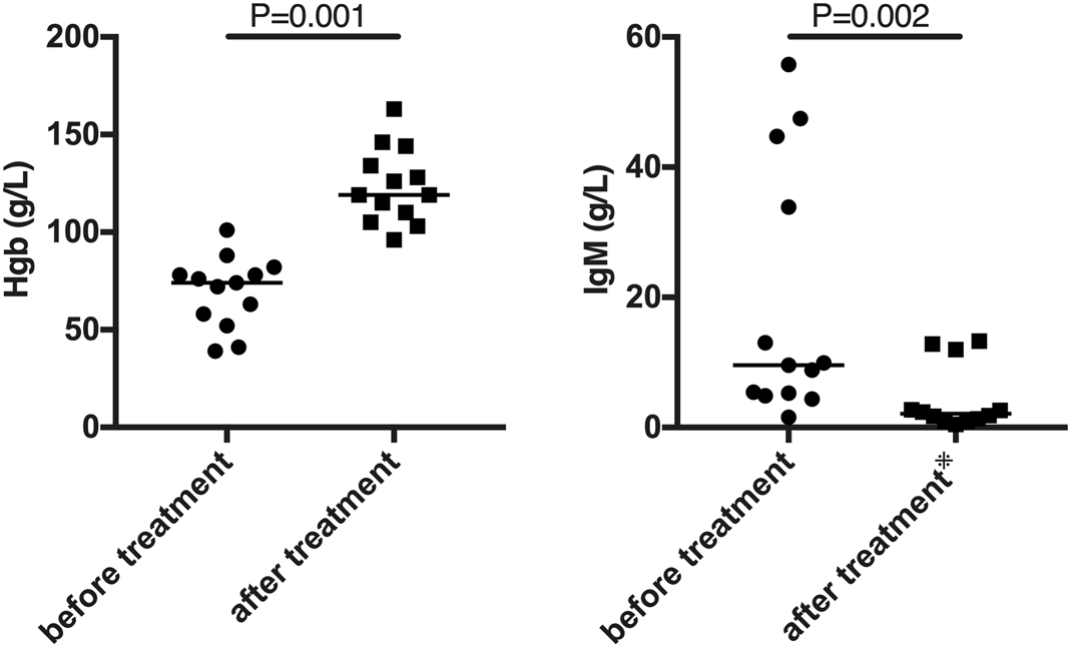
Laboratory response data in the responders. Hgb, haemoglobin; IgM, immunoglobulin M. ^※^n = 12; one responder did not have immunoglobulin M retested during follow-up. (Figure was performed on a personal computer using GraphPad Prism version 8.0.0 for Windows, GraphPad Software, San Diego, California USA, www.graphpad.com.)

The response data are shown in Fig. 1. Peripheral circulatory symptoms improved by definition in all responders and in 1 of 3 non-responders. Hgb levels increased by a median of 45 g/L among the responders. In the responders, median total bilirubin levels decreased from 30.4 μmol/L (range 6.2–154.2) to 17.1 μmol/L (range 6.4–31.7), median indirect bilirubin levels from 21.0 μmol/L (range 2.2–85.0) to 11.6 μmol/L (range 4.7–19.6), and median LDH levels from 280 U/L (range 124–517) to 242 U/L (range 147–517). One of the responders had a normal baseline IgM concentration and did not have IgM retested after treatment; the median IgM decreased from 7.11/L (range 1.58–55.8) to 2.10 g/L (range 0.48–13.27) among responders.

Among the non-responders, patients 6 and 16 were under treatment for 1 month and 2 months, respectively; patient 6 showed an improvement of anaemia-related symptoms, but the laboratory characteristics do not yet fulfil the criteria of PR. Patient 13 received supportive measures instead of second-line therapy during the study period, and his disease is now stable.

### Adverse events

The most common adverse event was neutropenia grade 3–4 (n = 3, 19%): patient 7 had a fever requiring treatment, and the other 2 patients had no evidence of infection (patients 12, 13). For infusion-related events with rituximab, anaphylaxis occurred in 1 patient presenting as hypotension, receiving DRC treatment (dexamethasone, rituximab and cyclophosphamide), and he responded to saline infusion and finished the rituximab infusion (patient 14).

### Follow-up

**Figure 2:**
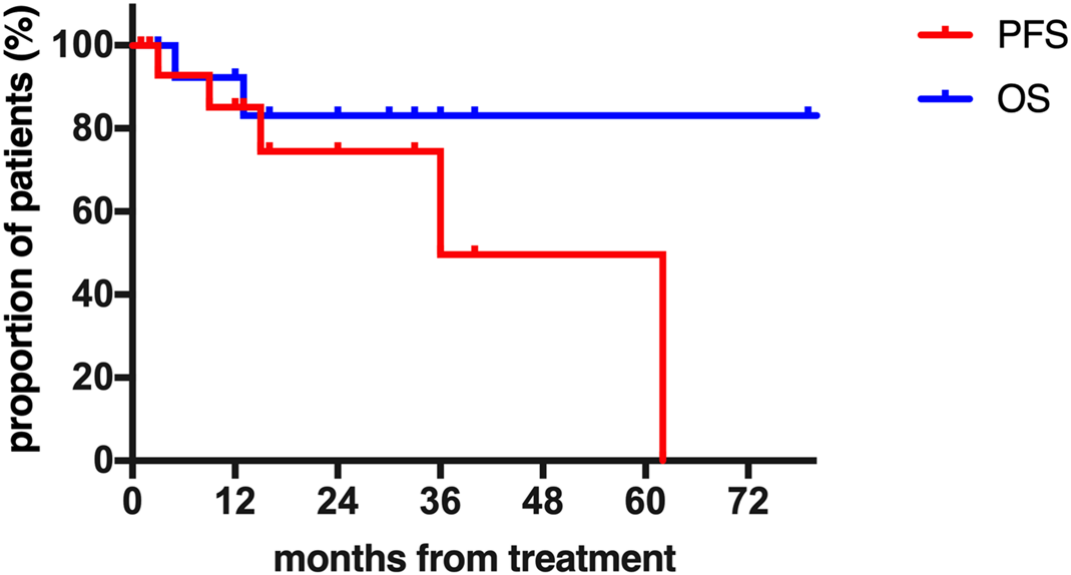
Overall survival and progression-free survival among all patients. (Figure was performed on a personal computer using GraphPad Prism version 8.0.0 for Windows, GraphPad Software, San Diego, California USA, www.graphpad.com.)

Median follow-up was 16 months (range 1–82), and the estimated median PFS was 36 months (Fig. 2). Five patients (31%; patients 1, 2, 7, 14, 15) experienced relapses, and 3 of them (patients 2, 7, 14) were treated with rituximab-containing therapy again. All 3 re-treatment patients underwent PR, and 1 of them fulfilled all CR criteria except the bone marrow examination (patient 14). Patient 1 died before further therapy could be administered. Patient 15 changed to Zanubrutinib, a novel inhibitor of Bruton tyrosine kinase, resulting in PR during the follow-up period.

Two patients died during follow-up. One responder (patient 1) died of disease progression 5 months after the initiation of treatment, and he did not have the chance to receive any further treatment. Patient 12 died of unknown reasons. The median OS was not yet reached.

## Discussion

CAD is a rare form of haemolytic anaemia, and only a few studies have been conducted regarding its treatment. The pathogenesis of CAD is recognized as a clonal lymphoproliferative B-cell disorder and a complement-mediated immune haemolytic anaemia^15^. In a Norwegian study, CAD-associated LPD was found to be distinct from LPL, MZL, and other previously recognized lymphoma entities, with the absence of an *MYD88*^L265P^ mutation^21^. However, the histological, immunophenotypic, and flow cytometric characteristics they described have not yet been confirmed by other studies. Our results show that some of the CAD patients had a clear diagnosis of WM or other indolent B-cell lymphoma, and the response was similar regardless of the concomitant LPD. The frequency of the *MYD88*^L265P^ mutation was quite low among the WM patients in our group. However, whether CAD is an independent LPD is still inconclusive.

As for the treatment of CAD, we have to adopt expert opinions rather than results from standardized clinical trials. According to the studies Berentsen’s group conducted, the rituximab plus bendamustine combination therapy and rituximab monotherapy may be used as a first-line treatment^15,17^. Bortezomib-based therapy and rituximab plus fludarabine therapy were proven efficacious in prospective trials and case reports^16,22–24^. In this study, we analysed any rituximab-containing therapy among CAD patients in one institution. The response rate was 81% altogether in 16 patients who had not previously received rituximab, which confirmed previous findings that rituximab either as a monotherapy or in combination is effective in treating CAD^13,14,16,17^. Two NRs were recently treated with a short follow-up time, and their treatment may need more time to take effect. Remissions lasted longer than 12 months, and the expected response duration was 36 months. Furthermore, although the number of patients included was limited, rituximab-based treatment would probably take effect when re-treatment is required.

When comparing our study with the bendamustine-rituximab combination trial^17^, our patients’ baseline Hgb and LDH levels were lower, and other baseline characteristics, inclusion criteria and response criteria were similar. The overall response rates were similar, although the CR rate of our study was remarkably lower and is even lower in reality, and the observed response duration was shorter mainly because of our shorter observation time. Regarding the safety of the therapy, our study shows acceptable outcomes. Thus, we think it reasonable to choose a rituximab-based therapy in accordance with individual patient characteristics, weighing the efficacy and tolerance of the treatment.

Inhibition of the classical complement pathway is another approach to treat CAD patients. Complement inhibitors, such as eculizumab, sutimlimab, ANX005 and APL-2, are under study, and the most approved option is sutimlimab^15^. Recently, it was reported that sutimlimab can rapidly stop C1*s* complement–mediated haemolysis, increase Hgb levels and preclude the need for transfusions^25^. Complement modulations have limitations that it will not improve cold-induced symptoms and require continuous administration indefinitely to maintain their effect^25^. However, novel complement-directed therapies can still be very promising in the future, especially in severe cases and acute exacerbations^18,26^.

This study has several limitations. First, it is a single-institution retrospective study, and the number of patients included in the study was limited, which might limit the generalizability of our results. An expanded sample size is required for further analysis. Second, the choice of treatment mainly depends on the experience of the clinicians, and the rituximab-based treatment used in our study had marked heterogeneity. Therefore, it is difficult to determine which specific regimen is better, and further prospective trials are required to optimize the therapy.

## Conclusion

In conclusion, our study shows a favourable effect of rituximab-containing therapy in patients with CAD.
